# The 9th Decade and Beyond: The Perils of the Narrative of Decline

**DOI:** 10.1093/geroni/igab046.2264

**Published:** 2021-12-17

**Authors:** Elaine Eliopoulos

**Affiliations:** University College London, University College London, England, United Kingdom

## Abstract

Twenty-three participants ranging in age from 80-102 years living on remote islands in the Pacific Northwest, USA reported the privileges of their current years. The aim of the study was to explore lived bodily experience and its impact on social exclusion. Participants utilized a unique visual methodology by photographing their experiences which highlighted daily life. While acknowledging that their years ‘before’ were different, and that life going forward may present unwelcome challenges, life in the now brought new joys and self-determination, despite various bodily compromises. Their perceptions of their bodies defied the dominant narrative of decline. These important findings warrant further investigation of the ways in which this emerging cohort views the challenges of aging bodies and their ability to remain socially connected. The role the dominant narrative of decline plays in their lives may prove to misdirect supports.

